# Localization for Dual Partial Discharge Sources in Transformer Oil Using Pressure-Balanced Fiber-Optic Ultrasonic Sensor Array

**DOI:** 10.3390/s24144450

**Published:** 2024-07-10

**Authors:** Feng Liu, Yansheng Shi, Shuainan Zhang, Wei Wang

**Affiliations:** School of Electrical and Electronic Engineering, North China Electric Power University, Beijing 102206, China

**Keywords:** partial discharge, pressure-balanced fiber-optic ultrasonic sensor, fault localization, dual partial discharge sources detection

## Abstract

The power transformer is one of the most crucial pieces of high-voltage equipment in the power system, and its stable operation is crucial to the reliability of power transmission. Partial discharge (PD) is a key factor leading to the degradation and failure of the insulation performance of power transformers. Therefore, online monitoring of partial discharge can not only obtain real-time information on the operating status of the equipment but also effectively predict the remaining service life of the transformer. Meanwhile, accurate localization of partial discharge sources can assist maintenance personnel in developing more precise and efficient maintenance plans, ensuring the stable operation of the power system. Dual partial discharge sources in transformer oil represent a more complex fault type, and piezoelectric transducers installed outside the transformer oil tank often fail to accurately capture such discharge waveforms. Additionally, the sensitivity of the built-in F-P sensors can decrease when installed deep within the oil tank due to the influence of oil pressure on its sensing diaphragm, resulting in an inability to accurately detect dual partial discharge sources in transformer oil. To address the impact of oil pressure on sensor sensitivity and achieve the detection of dual partial discharge sources under high-voltage conditions in transformers, this paper proposes an optical fiber ultrasonic sensor with a pressure-balancing structure. This sensor can adapt to changes in oil pressure environments inside transformers, has strong electromagnetic interference resistance, and can be installed deep within the oil tank to detect dual partial discharge sources. In this study, a dual PD detection system based on this sensor array is developed, employing a cross-positioning algorithm to achieve detection and localization of dual partial discharge sources in transformer oil. When applied to a 35 kV single-phase transformer for dual partial discharge source detection in different regions, the sensor array exhibits good sensitivity under high oil pressure conditions, enabling the detection and localization of dual partial discharge sources in oil and winding interturn without obstruction. For fault regions with obstructions, such as within the oil channel of the transformer winding, the sensor exhibits the capability to detect the discharge waveform stemming from dual partial discharge sources. Overall, the sensor demonstrates good sensitivity and directional clarity, providing effective detection of dual PD sources generated inside transformers.

## 1. Introduction

Transformers are common high-voltage power equipment, characterized by a large installation base and high economic value. Their safe and stable operation directly affects the reliability of the power grid. Among the various fault types, insulation failure in transformers is one of the most common, making online monitoring and fault diagnosis of transformer insulation status paramount [1]. Partial discharge (PD) occurs in the insulated portions of electrical equipment under prolonged high-voltage conditions, manifesting as localized discharges. These discharges often originate from various defects within the insulation media, such as minute cracks, hidden bubbles, or accumulations of metallic impurities. The discharge process not only generates gases and chemical substances but also significantly elevates local temperatures, leading to adverse effects like corrosion on insulation materials. These consequences accelerate the degradation process at the insulation defects, thereby shortening the service life of the equipment [2]. Therefore, the monitoring and prevention of partial discharges are crucial steps in ensuring the safe and stable operation of power equipment.

Partial discharge testing is an effective method to evaluate the PD level in transformers, accurately reflecting the health status and insulation aging degree of high-voltage electrical equipment [3]. In large transformers with high voltage ratings, due to their compact internal structure, high and unevenly distributed electric field strengths, the discharge types are more complex, often leading to simultaneous discharges from multiple PD sources [4]. These discharges, with their superimposed waveforms, become more intricate, making it challenging for traditional external sensors to detect and distinguish such discharge types. Even if the simultaneous discharge waveforms of multiple PD sources are captured, they are difficult to differentiate from environmental interference waveforms. These issues increase the difficulty for maintenance personnel, and missed detections or repairs can result in poor maintenance outcomes for power equipment, while repeated maintenance inspections further increase transformer maintenance costs. Overall, research on multi-point detection and localization techniques for internal PD in transformers is crucial for enhancing the accuracy and efficiency of fault detection and allowing maintenance personnel to optimize maintenance plans [5]. Therefore, an in-depth study of the discharge characteristics of dual PD sources under complex internal conditions in large transformers, the accurate identification of discharge types, and the precise localization of PD areas are significant for timely detecting potential faults, predicting the remaining life of equipment, and developing maintenance plans [6]. This will contribute to ensuring the safe and stable operation of power systems.

During the occurrence of partial discharge, it is often accompanied by phenomena such as pulse current, radiation light, and sound. These characteristic signals contain information about the energy intensity and positional direction of the PD sources [7]. Through digital signal processing techniques and the application of corresponding algorithms, quantitative analysis of PD phenomena can be achieved. This holds significant academic value and practical implications for the in-depth study of PD mechanisms and the guidance of fault inspection and repair work. Currently, there are two major categories of PD localization techniques: electrical signal localization and acoustic signal localization. Depending on the different characteristic quantities, they can be further divided into the pulse current method, the ultra-high frequency method, and the ultrasonic localization method. As an effective and accurate localization method, acoustic methods have been widely used in the industry, and many scholars have conducted extensive research on this method. The general acoustic localization method employs a piezoelectric transducer array placed on the outer wall of the transformer oil tank. It utilizes the time difference of ultrasonic signals reaching different sensors to solve equations and determine the location of the partial discharge source. Depending on the adopted reference time scale, it can be further divided into “acoustic-acoustic” and “electric-acoustic” methods [8,9]. This method is cost-effective and relatively easy to install, but ultrasonic waves undergo attenuation and distortion after reflection from the transformer oil tank. Additionally, the external environment of the oil tank is prone to signal interference, resulting in a low signal-to-noise ratio [10]. Therefore, the distributed installation of sensors is required to achieve high localization accuracy. The received ultrasonic signals are scattered, and the signals received by different sensor elements vary significantly, which can easily lead to the non-convergence of the algorithm’s solution. In particular, for dual PD sources occurring inside the transformer, traditional piezoelectric transducers cannot be expected to clearly distinguish and accurately locate them. The Fabry–Perot (F-P) sensor utilizes the ultrasonic signals generated by PD to deform the silica diaphragm located at the probe [11]. By analyzing the optical changes within the F-P cavity, fault detection of PD can be achieved. This fiber-optic ultrasonic sensor, with its optical signal as the signal transmission medium, boasts excellent insulation properties and can be built into the transformer’s interior [12,13]. Currently, most of these built-in detection devices are installed at the oil change valve at the bottom of the transformer oil tank. However, under the influence of oil pressure, the sensitivity of the sensing diaphragm decreases, and the sensor cannot fully respond to dual PD signals. This limits its detection range and fails to provide accurate partial discharge detection for the transformer’s overall, especially the winding section located at the bottom.

To address the aforementioned issues, this paper presents a pressure-balanced optical fiber ultrasonic sensor. By introducing perforation, transformer oil can enter the F-P interference cavity, thus minimizing the impact of oil pressure on the diaphragm’s sensitivity. However, the ingress of transformer oil can reduce the reflectivity of light. To counter this, we have implemented an alternating coating of SiO_2_ and Ta_2_O_5_ on the silica diaphragm and fiber end face, enhancing reflectivity and mitigating the interference caused by transformer oil in the F-P interference cavity. Utilizing light as the signal transmission medium, this sensor boasts a high insulation level, enabling the detection of dual PD ultrasonic signals from the bottom of transformer oil tanks. The collected PD ultrasonic signals from the sensor array are processed using a direction-finding cross-localization algorithm, accurately identifying and precisely locating the dual PD sources within the tank. Experimental evaluations conducted on a 35 kV single-phase transformer at various locations have demonstrated the effectiveness and precision of this method in detecting and locating dual PD sources in transformer winding interturn. However, for PD sources within the oil channels, the method offers qualitative detection but fails to achieve precise localization.

In order to detect the discharge fault of dual partial discharge sources in transformer oil, this paper designs an optical fiber ultrasonic sensor with a pressure balance structure, which allows transformer oil to enter the sensor cavity through drilling. In order to minimize the influence of transformer oil on the sensitivity of the sensor, the reflectivity of the silica diaphragm and the fiber end face is improved by coating. The well-fabricated sensors are assembled into a sensor array, and the method of cross-direction measurement is used to locate the fault position of the dual partial discharge sources and detect the discharge phenomenon of dual partial discharge sources at different positions of the transformer.

**Figure 1:**
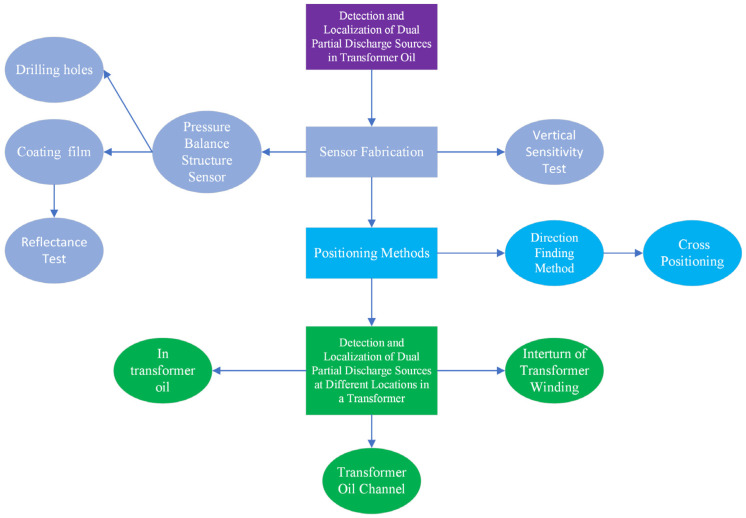
Writing organization structure diagram.

In summary, the combination of the pressure-balanced ultrasonic sensor array and the direction-finding cross-localization algorithm exhibits excellent performance in detecting and locating dual PD sources in transformer oil tanks, particularly in scenarios without obstacles. This approach provides a novel solution and research direction for localizing complex PD faults in high-voltage power transformers. The organizational structure of this writing is illustrated in Figure 1. The first-level purple area represents the overall purpose of this paper, which is to detect and locate the discharge phenomena of dual partial discharge sources in transformer oil. The second-level gray area covers the sensor fabrication section, corresponding to Section 2 of the paper. In this section, we have developed sensors that can adapt to the oil pressure of transformers. The improvements focus on two main points: the first is drilling holes in the sensor cavity to allow transformer oil to enter, and the second is coating the sensor to enhance its reflectivity. Finally, we conducted vertical sensitivity tests using the fabricated sensors. The third-level blue section represents the localization method, which corresponds to the content of Section 3 in the article. The first step is to determine the direction of the partial discharge sources, and the second step is to use the results of the directional measurements to perform cross-localization. The fourth-level green area represents the experiments we conducted for detecting and localizing dual partial discharge sources at different locations within the transformer, specifically in three representative positions: inside the transformer oil, within the transformer oil channel, and at the interturn of the transformer winding. These locations have varying degrees of obstruction to the ultrasonic waves.

## 2. Pressure-Balanced Fiber-Optic Ultrasonic Sensor and Dual PD Localization System

**Figure 2:**
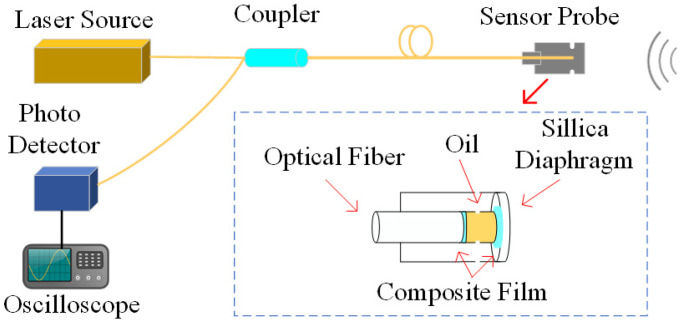
Structure of the dual PD detection system.

The structure of the pressure-balanced fiber-optic ultrasonic sensor is illustrated in Figure 2. The dual PD source localization system comprises a sensor probe, laser source, coupler, and photodetector, and they are interconnected via a single-mode fiber (SMF). The sensor probe features a small hole at the F-P cavity, allowing transformer oil to enter the cavity, thereby minimizing the hindrance of oil pressure on diaphragm vibrations. However, the influx of transformer oil can impact the optical performance of the F-P cavity. To address this, the inner surface of the silica diaphragm is coated with a total reflection film, while the fiber end face is coated with an optical dielectric film composite of Ta_2_O_5_ and SiO_2_. Ta_2_O_5_ is an insulating material with stable molecular properties, exhibiting exceptional thermal and chemical stability, high voltage resistance, a high refractive index, and a high transmission coefficient. Ta_2_O_5_ coatings are commonly used for high-refractive-index thin-film materials [14]. SiO_2_, another insulating material with a high refractive index, boasts a melting point of up to 1723 °C, chemical inertness, and high voltage resistance [15]. By employing an alternating coating process with these two materials, the sensor achieves both high reflectivity and excellent adhesion, leading to a long service life. In contrast to the metal material coating solution, this sensor design does not create a floating potential, ensuring no impact on the internal electric field distribution of the transformer.

The laser source emits monochromatic light that propagates along SMF and enters the F-P cavity. According to the Huygens–Fresnel principle, the reflectivity of the incident light at the interface between the fiber end face and air is approximately 3.6%, while the remaining 96.4% of the incident light passes through the interface and enters the F-P cavity, striking the silica diaphragm. Most of this light transmits through the silica diaphragm, and the remaining 3.3% reflects back into the fiber at the inner surface of the silica diaphragm. The ultrasonic signal generated by partial discharge reaching the sensor diaphragm causes vibrational deformation of the diaphragm, resulting in a slight change in the length of the F-P cavity [16]. This change triggers an interference pattern between the two beams of light, leading to a change in light intensity. By detecting this change in light intensity through the circulator port and converting it into an electrical signal detectable by an oscilloscope using a photodetector, the interference-modulated light intensity can be expressed as follows:

(1) $$I_{r}(l)=\frac{r_{1}+r_{2}-2\sqrt{r_{1}r_{2}}\cos\delta }{1+r_{1}r_{2}-2\sqrt{r_{1}r_{2}}\cos\delta }I_{0}(l)$$

(2) $$\delta =\frac{4\pi ∆l}{\lambda }$$

In the expression, *l* represents the length from the fiber end face to the inner surface of the silica diaphragm, which is also known as the length of the F-P cavity. I_0_(*l*) denotes the incident light intensity (the unit is mW/m^2^), which remains constant when there is no external signal, as the F-P cavity length remains unchanged. *λ* is the wavelength of the laser light source (the unit is μm). *r*_1_ represents the reflectance at the fiber end interface, while *r*_2_ represents the reflectance at the inner surface of the silica diaphragm. *δ* is the phase difference between adjacent beams (the unit is rad); Δ*l* is the optical path difference between the two interfering light beams (the unit is μm). With fixed reflection coefficients *r*_1_ and *r*_2_, the intensity of the interference light after the reflection of the two beams is solely dependent on the F-P cavity length (*l*) of the sensor.

When external vibration signals act on the sensor, they cause the diaphragm to undergo bending deformation, altering the cavity length of the sensor and subsequently changing the intensity of the interference light between the two beams. By demodulating the received optical intensity signal, the measurement of the partial discharge ultrasonic signal can be achieved.

**Figure 3:**
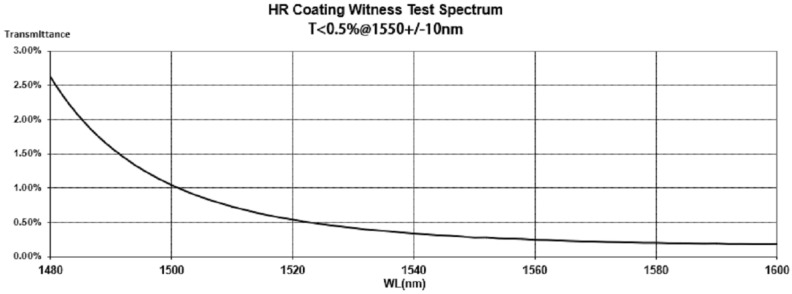
Reflectivity curve of the inner surface of the quartz film after coating.

The cladding diameter of the single-mode fiber used in the pressure-balanced fiber-optic ultrasonic sensor is 125 μm. The coating area must cover the entire region where the incident light beam strikes the inner surface of the silica diaphragm. To minimize the impact on the vibration of the silica diaphragm, the coating radius should be as small as possible, while also considering potential deviations during the welding of the silica diaphragm and the sensor cavity during the manufacturing process. Therefore, we deposited a circular film with a diameter of 500 μm and a thickness of 260 nm at the center of the inner surface of the silica diaphragm, which has a diameter of 3.5 mm and a thickness of 30 μm. Considering economic costs, we did not opt for an expensive, fully reflective film. Instead, combining engineering practicality, this article adopts a cost-effective coating with a relatively high reflectance. After coating the inner surface of the silica diaphragm, we tested its refractive index, and the results are shown in Figure 3. The laser source used in the partial discharge detection system designed in this article has a center wavelength of approximately 1550 nm. According to the reflectance curve, it can be seen that the reflectance of the inner surface of the silica diaphragm after coating is approximately 99.7%.

Given the technical challenges of coating the fiber end face and the fact that the F-P interference cavity relies on transmitted light, a comprehensive consideration of the relationship between reflectance and transmittance is essential. In this paper, the reflectance of the fiber end face after coating is set at 20%, with a transmittance of 80%. The processed sensor was placed in transformer oil for 48 h to allow the oil to completely penetrate into the F-P cavity. Utilizing the MS9740A spectrometer (The MS9740A spectrometer is manufactured by Anritsu in Atsugi, Japan, and we purchased it from Shanghai, China), its optical power was measured at 534.6 μW, with a reflectance of 19.8%. Compared to the 3.6% reflectance without coating, this method significantly enhances the overall performance of the sensor after transformer oil enters the F-P interference cavity. Considering the practical application of placing the sensor at different depths inside the transformer, this paper designed an experiment to investigate the vertical sensitivity of the sensor in transformer oil. The vertical sensitivity characteristic refers to the variation in the maximum voltage amplitude of the sensor’s output waveform at different depths in the transformer oil.

**Figure 4:**
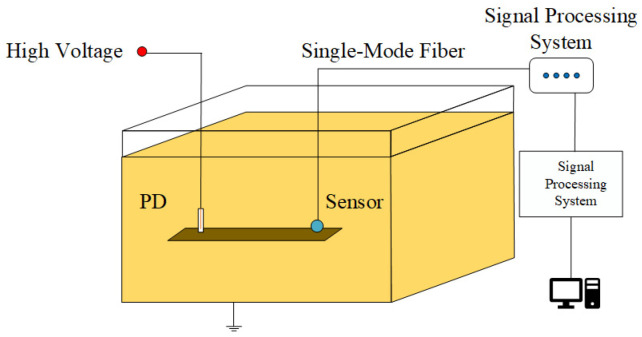
Layout diagram for vertical sensitivity test of sensor.

**Figure 5:**
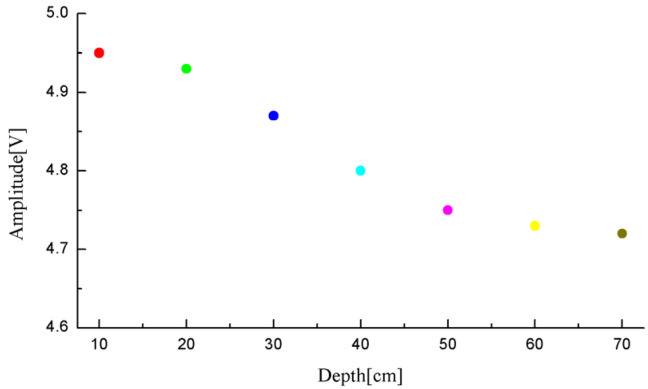
Vertical sensitivity decay curve of the sensor.

The sensor was fixed on an insulating wooden strip with a needle-plate electrode, maintaining a spacing of 60 cm. The probe of the sensor was positioned directly facing the needle-plate electrode. Both the sensor and the needle-plate electrode were submerged into the transformer oil at depths ranging from 10 cm to 70 cm, as depicted in Figure 4. During each experiment, the maximum value of the waveform voltage was selected, and the experimental results are presented in Figure 5. It is worth noting that the sensors used in this study were not precisely manufactured by a factory with uniform specifications. Instead, after the components were processed, they were manually cut and welded in our laboratory, resulting in significant variations in sensor sensitivity. Furthermore, considering the inherent randomness of simulating partial discharge in insulating oil, the discharge magnitude was not a fixed value, leading to certain deviations in ultrasonic energy. Therefore, Figure 5 can only qualitatively demonstrate the influence of oil pressure on the sensitivity of a single sensor.

**Figure 6:**
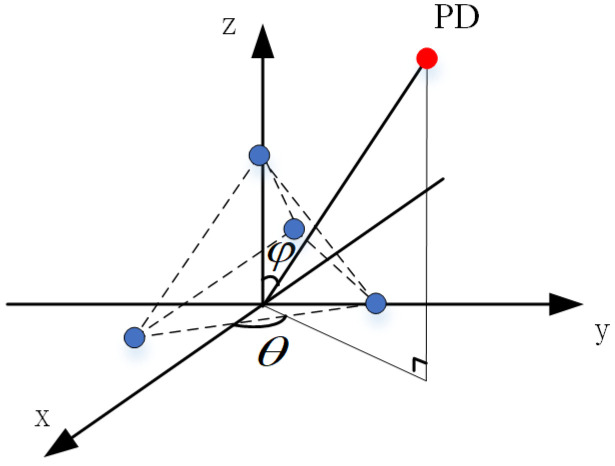
Spatial structure diagram of sensor array.

Overall, as the depth increases and the oil pressure rises, the viscous coefficient acting on the sensor diaphragm increases, subsequently elevating the vibrational resistance, resulting in a decrease in sensor sensitivity. Based on this pressure-balanced structure sensor, this study has further designed a sensor array for the localization of dual PD sources. According to phased array theory, to mitigate the radiation energy occupied by grating lobes and enhance the array antenna gain, the element spacing should be less than or equal to half of the wavelength of the target being measured. Additionally, to guarantee a sufficiently large effective area for signal reception and a high resolution, half of the wavelength is chosen as the element spacing. In the experiments conducted in this paper, the propagation velocity of ultrasonic waves in oil is 1420 m/s, with a central frequency of 30 kHz, resulting in a wavelength of 47.33 mm. Moreover, to ensure a sufficiently large receiving surface for the array and similar signal reception among elements, thereby avoiding positioning errors caused by inconsistent element responses, the array layout was optimized through multiple experiments. Ultimately, a regular tetrahedron structure with an element spacing of 20 mm was adopted. Under these parameters, the pressure-balanced fiber-optic ultrasonic sensor array exhibits excellent signal acquisition performance. The spatial structure diagram of the array is presented in Figure 6, where *θ* and *ϕ* represent the azimuth and elevation angles of the PD source, respectively. The method of establishing the three-dimensional Cartesian coordinate system for the sensor array is also applicable to subsequent discussions and calculations involving PD source direction measurements in this paper. The technical changes of the sensors in this section are summarized in Table 1.

## 3. Method for Locating Dual PD Sources

The Multiple Signal Classification (MUSIC) algorithm is a high-resolution spectral estimation technique utilized in sensor array signal processing. It relies on the orthogonality principle between signal subspaces and noise subspaces and determines the direction of signals by searching for the peaks in the spatial spectrum [17]. As a result, MUSIC is a direction-finding method that is based on the energy of sound sources.

### 3.1. The Principle of Direction-Finding with the MUSIC Algorithm

Based on the principle of spatial spectrum estimation, assuming *X*(*t*) represents the received signal at the array, its covariance matrix is computed as follows:

(3) $$R=E\left\{X\left(t\right)X{\left(t\right)}^{H}\right\}=A(\varphi ,\theta )R_{S}A^{H}(\varphi ,\theta )+R_{N}$$

In the expression, *R* represents the covariance matrix of the array signal, while *A*(*φ*, *θ*) is the steering vector of the signal, encoding the direction of arrival (DOA) of the PD source [18]. Specifically, *θ* denotes the azimuth angle, and *φ* represents the elevation angle of the PD source, as illustrated in Figure 3. *R_S_* and *R_N_* are the signal covariance matrix and noise covariance matrix, respectively. By performing an eigendecomposition on the array signal covariance matrix *R*, a function involving the noise subspace *U_N_* can be obtained. According to the properties of subspaces, the signal steering vector is orthogonal to the noise subspace. However, in practical scenarios, noise is always present, and thus the orthogonality is not perfect. The actual DOA estimation is achieved by evaluating all possible DOA values and identifying the minimum value. This minimum-finding process can be transformed into a maximum-seeking problem through a reciprocal relationship.

(4) $$P_{music}=\frac{1}{A^{H}\left(\varphi ,\theta \right)U_{N}U_{N}^{H}A\left(\varphi ,\theta \right)}$$

*P_music_* is a spatial spectrum function, serving as a calculation formula for energy. The energy value of ultrasonic signals at the location of partial discharge is significantly higher than that at non-partial discharge locations where only sound wave reflections are present. For a single PD source, after searching through the entire spatial spectrum, the MUSIC algorithm identifies the peak with the highest energy. The corresponding values of *φ* and *θ* at this peak represent the directional information of the PD source. This is the foundation of the MUSIC algorithm for DOA finding. Even in the case of continuous discharge from a single point, the location of the energy extremum in the spatial spectrum remains unchanged, with only one peak representing the direction of the partial discharge source. Therefore, it can be distinguished from the case of simultaneous discharge from dual PD sources. By constructing a spatial spectrum function, the MUSIC algorithm enables DOA estimation of PD sources, offering better resolution compared to traditional beamforming methods. This allows for direction finding of dual PD sources signals simultaneously.

### 3.2. Localization Algorithm

**Figure 7:**
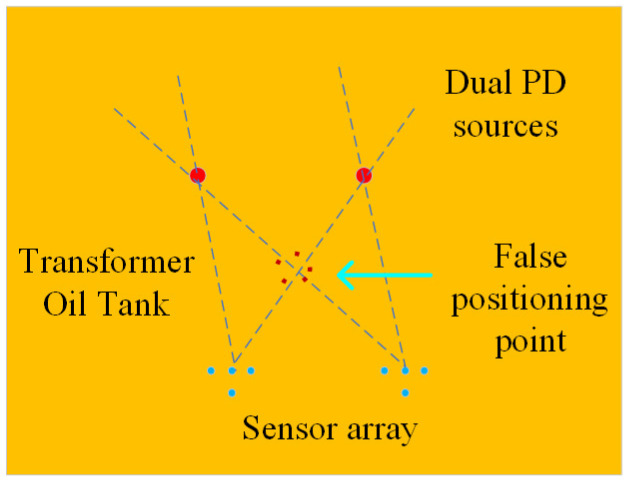
Schematic diagram of cross location for dual PD sources direction finding.

The fundamental principle of the direction-finding cross-localization algorithm relies on two direction-finding stations, each comprising sensor arrays, to determine the DOA of a common target. Subsequently, through spatial geometric calculations, the coordinate position of the target is accurately determined, as depicted in Figure 7. This approach enables precise localization based on the intersection of the DOA measurements from the two stations.

As depicted in the localization schematic diagram in Figure 6, when the sensor arrays and the partial discharge location are situated on the same horizontal plane, theoretically, the localization method may encounter false positioning points. However, taking into account the actual direction-finding errors, the occurrence of false positioning points is rare. Furthermore, considering the redundancy inherent in equipment maintenance plans, maintenance personnel tend to prioritize avoiding missed fault detections during practical inspections. Therefore, in the subsequent discussions of this paper, the scenario of false positioning points will be disregarded.

## 4. Experimental Testing and Result Analysis

The dual PD sources localization testing platform for transformer oil comprises a transformer oil tank, a sensor array, a high-voltage pulse generator, and two needle-plate electrode discharge tubes simulating partial discharges. The transformer oil tank has dimensions of 200 cm in length, 100 cm in width, and 150 cm in height. It is filled with 25# high-voltage electrical insulation oil, and the outer shell of the tank is grounded. To simulate the fault scenario of simultaneous discharges from dual PD sources, we connect the two discharge tubes in series to the high-voltage pulse generator. By adjusting the spacing between the two needle-plate electrodes and through multiple voltage pressurization attempts, under the excitation of a 20 kV high-voltage pulse, the two series-connected needle-plate electrodes can simultaneously undergo breakdown and discharge, and the discharge waveform can be detected through an oscilloscope.

### 4.1. Detection Experiment of Dual PD Sources in Transformer Oil

As depicted in Figure 6, a spatial Cartesian coordinate system is established, connecting the PD sources to the origin. Through spatial geometry knowledge, the DOA estimation of the acoustic sources can be obtained, specifically the azimuth angle and the elevation angle. Furthermore, to evaluate the performance of the dual PD sources localization system, it is imperative to define the distance error between the actual PD source position and the theoretically estimated position. Therefore, the spatial distance between the actual PD source’s position and the estimated position is defined as the distance error. This definition holds true for the subsequent error analysis presented in the following sections.

**Figure 8:**
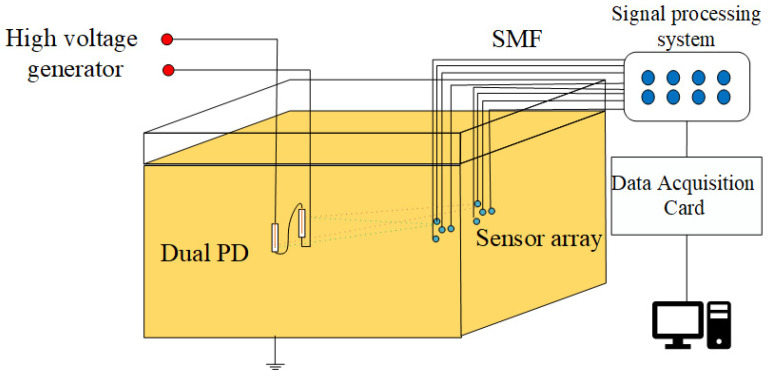
Layout diagram for the localization experiment of dual PD sources in transformer oil.

With the right bottom corner of the transformer oil tank serving as the origin, a comprehensive spatial Cartesian coordinate system is established. Two sensor arrays are deployed inside the tank at positions (45 cm, 10 cm, and 50 cm) and (55 cm, 10 cm, and 50 cm), respectively. To test the overall localization performance of the system for PD sources at different locations, the oil tank is divided into three zones, and localization experiments are conducted sequentially in each zone. Figure 8 provides a top-view of the layout of the dual PD sources positions, while Figure 8 illustrates the experimental setup.

**Figure 9:**
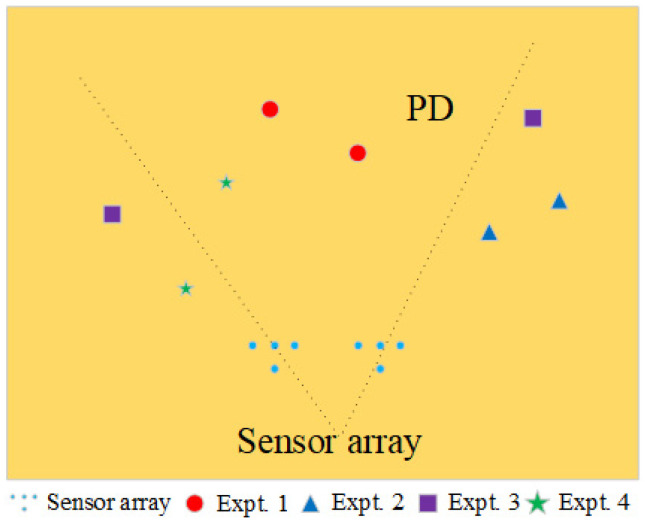
Aerial view of the layout of dual PD sources.

**Figure 10:**
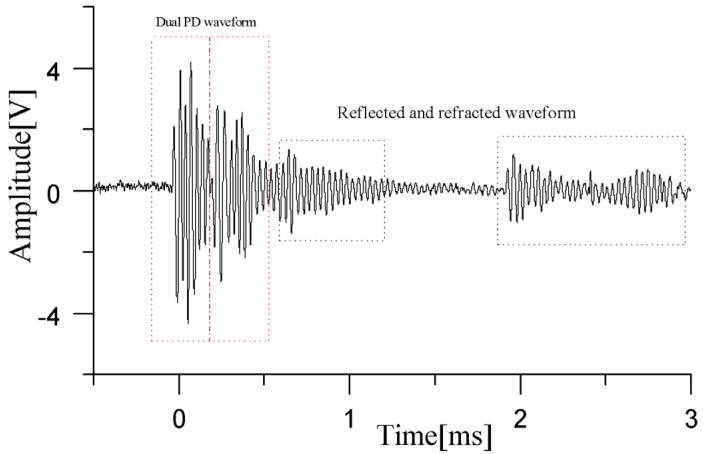
Ultrasonic waveform of dual PD sources in transformer oil detected by the sensor.

In the first experiment, two PD sources were placed at 47 cm, 48 cm, and 47 cm and 50 cm, 27 cm, and 53 cm, corresponding to the circular positions in Figure 9. Upon the occurrence of partial discharges, the waveforms detected by the sensors are presented in Figure 10. As observed from the waveform plots, the pressure-balanced fiber-optic ultrasonic sensors were able to accurately detect the discharge waveforms from both PD sources. Based on the signals detected by the sensor array and processed through a direction-finding and cross-localization algorithm, the estimated positions were determined as 42.7 cm, 58.8 cm, and 41.2 cm and 48.2 cm, 21.1 cm, and 48.6 cm, with distance errors of 12.9 cm and 7.6 cm, respectively.

Subsequently, following the methodology of the first experiment, the dual PD sources were placed at different locations. In the second experiment, the two PD sources were located on the same side of the sensor array, corresponding to the triangular positions in Figure 9; in the third experiment, the PD sources were positioned on opposite sides of the two arrays, at the square positions in Figure 9; and in the fourth group of experiments, the partial discharge source is located at the position of the star in Figure 9, respectively, to verify the positioning accuracy when the deviation between the dual PD sources is significant. The experimental results are presented in Table 2, which indicates that the positioning accuracy of the sensor array with a pressure-balanced structure generally meets engineering requirements, with the maximum distance errors being 17.7 cm and 13.1 cm in the second experiment with the triangular layout. This is attributed to the fact that in this region, there are more overlapping areas between the waveforms of the two PD sources, resulting in less distinct peak amplitudes of the direct waves, thereby causing a larger error in solving the energy extremum using the localization algorithm. In contrast, the circular layout in the first experiment exhibited the smallest positioning error, with distance errors of 12.9 cm and 7.6 cm. This is because in this region, the sensor array has the best directivity, receiving signals with higher energy and better sensitivity. Considering that only four sensors were used to form the array in this study, using a larger array with more sensors to cover a broader reception area would result in the reception of richer partial discharge signals, further enhancing the positioning accuracy for dual PD sources.

**Figure 11:**
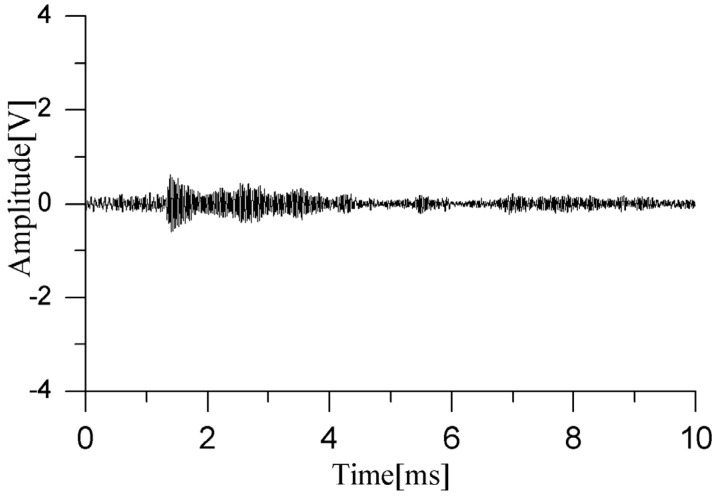
Ultrasonic waveform of dual PD sources after adding insulating pressboard.

In practical scenarios, if the dual PD sources are located inside the transformer winding and the sensor array is arranged outside the windings within the oil tank, the ultrasonic signals will encounter obstructions from the insulating pressboard. These adverse factors can increase the difficulty of detecting partial discharge signals. To simulate this situation, a 60 cm × 100 cm, 2 mm thick insulating pressboard was inserted between the sensor array and the PD sources. To more realistically mimic the internal environment of a transformer, the insulating pressboard was fully soaked with transformer oil beforehand. Through repeated experiments, it was found that the sensors had difficulty responding in the presence of such obstacles. In rare cases, partial discharge waveforms could be detected, as shown in Figure 11, but the waveforms that penetrated the pressboard were mixed with diffracted waves bypassing the insulating pressboard, resulting in very low voltage amplitudes and making it impossible to distinguish between direct waves and diffracted waves.

Next, further experimental studies will be conducted to investigate the impact of transformer winding on the detection of dual partial discharge sources.

### 4.2. Detection Experiment of Dual PD Sources in the Transformer Oil Channel

**Figure 12:**
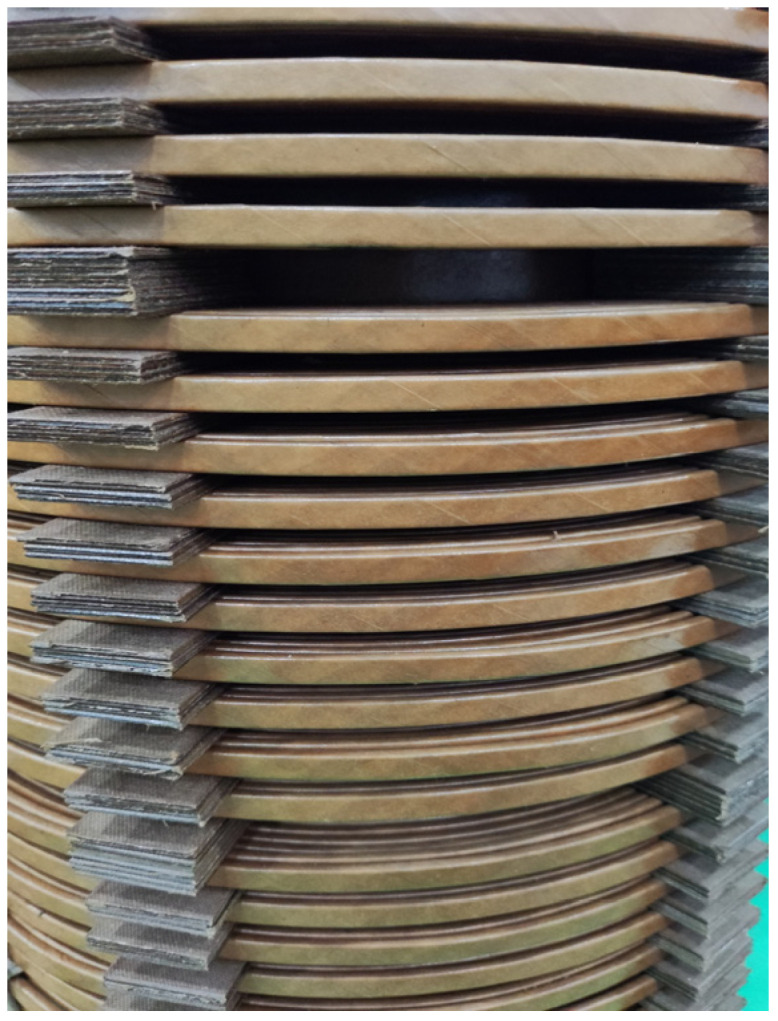
Physical diagram of transformer winding model.

**Figure 13:**
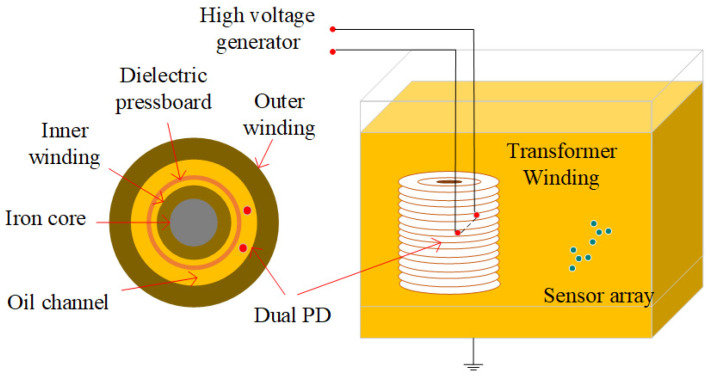
Layout diagram for the experiment of dual PD sources in transformer oil channel.

To verify the accuracy of the partial discharge localization system, we further conducted experiments using a 35 kV single-phase transformer winding model. This model consists of inner and outer windings with oil ducts in-between and 2 mm thick insulating pressboards. Detailed parameters are presented in Table 3. A realistic image of the windings is shown in Figure 12. The experimental setup for the localization of dual partial discharge sources in the transformer oil channel is depicted in Figure 13.

**Figure 14:**
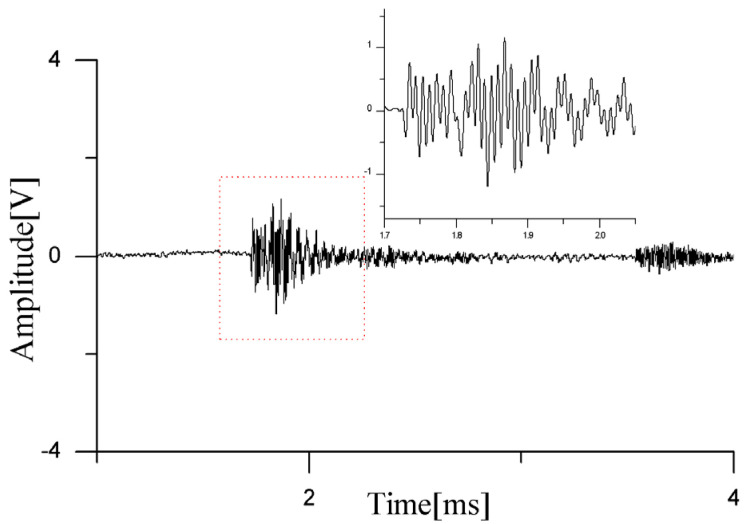
Ultrasonic waveform of dual PD sources in transformer oil channel.

When dual partial discharge occurs in the transformer oil channel, the ultrasonic signal needs to pass through the gap between the outer windings to reach the sensor array inside the oil tank. The waveform of the dual partial discharge detected by the sensor is shown in Figure 14. It can be seen that the ultrasonic wave can pass through the gap between the winding to reach the sensor array, but the signal amplitude is attenuated, and due to the blocking of the winding, the waveform is distorted and does not show the normal oscillating attenuation trend. Such a waveform cannot be used for actual engineering localization.

### 4.3. Detection Experiment for Dual PD Sources in Transformer Winding Interturn

**Figure 15:**
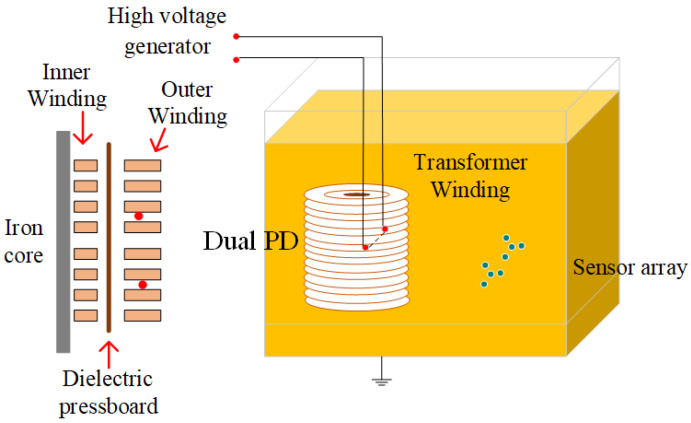
Experiment on locating dual PD in transformer winding interturn.

The interturn of transformer windings refers to the uniform gap between stacked winding cakes of different layers, which are separated by insulating paper and insulating cardboard. Due to poor manufacturing processes and the introduction of impurities during later operation of the transformer, it can lead to local electric field concentration, causing partial discharge and resulting in interturn short-circuit of the winding coil [19]. This is a common fault type in transformer windings. When a relatively minor interturn short-circuit occurs in the winding, the transformer can still operate. However, if it continues to operate with the fault for a long time, it may lead to more severe consequences or even damage the transformer [20]. Multiple partial discharges can accelerate the speed of insulation degradation and increase the risk of an interturn short circuit. The experimental layout for testing the dual partial discharge sources in transformer winding interturn is shown in Figure 15.

**Figure 16:**
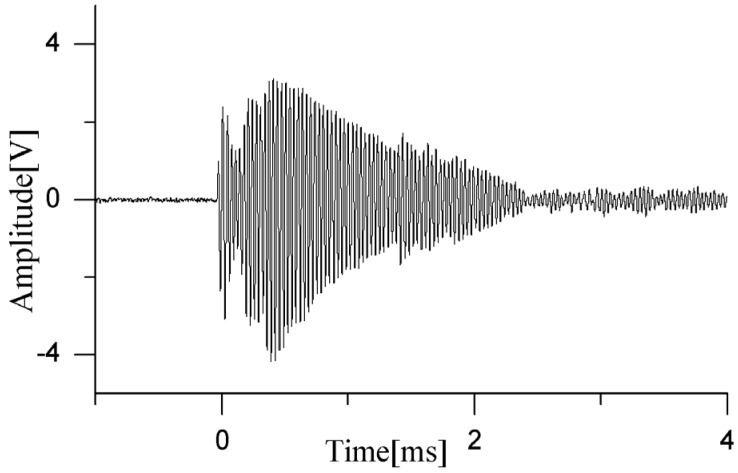
Ultrasonic waveform of dual PD sources in transformer winding interturn.

When dual partial discharge sources are arranged between the turns of the outer winding, a partial discharge waveform is detected by the sensor, as shown in Figure 16, and the localization results are presented in Table 4.

As seen from the signals received by the sensor in Figure 15, when the dual PD sources are located in the winding interturn, the ultrasonic signals emitted by the PD sources are not blocked by the windings. The first few cycles of the waveform can propagate through the gaps of the winding to the sensor array without distortion. Due to the refraction and reflection of the ultrasonic waves by the winding gaps, the latter half of the signal exhibits more reverberations. The amplitudes of these interfering waveforms are significantly lower than those of the direct wave signals. From an energy perspective, these interferences may increase the number of energy peaks, but they do not affect the selection of the first and second extreme values, which represent the positional information of the two sound sources. Therefore, by arranging four sensor arrays on both sides, it is possible to achieve the localization of dual partial discharge sources occurring in the interturn space and on the surface of the transformer windings.

Our method for detecting dual partial discharge sources involves placing sensors inside the transformer, which differs from the approach of installing sensors outside the transformer’s metal tank. The latter method is unable to detect the simultaneous discharge of dual partial discharge sources due to ultrasonic attenuation and interference from external signals. The built-in detection method offers high accuracy but faces two major challenges. The first is that the sensors need to overcome the decrease in sensitivity caused by the pressure of the transformer’s insulating oil, which is an objective technical issue that our improved sensors aim to address. The second challenge is a subjective concern stemming from manufacturers and users’ worries about this invasive detection method. The more sensors used, the greater their concern. Currently, there are few algorithms suitable for simultaneous localization of dual sound sources. Currently, we can only use a single sensor array for direction finding and cross-check the results from two arrays to achieve localization, which is a cumbersome and complex method and a last resort as we have not found a better solution yet. A sensor array requires four sensors, and two arrays would need eight sensors. To detect and locate partial discharges on both sides of a single-phase winding, the number of sensors required could reach up to 16. Therefore, the current localization method of the paper can only be implemented under laboratory conditions, and it will be of engineering practicability only after reducing the number of sensors used. Therefore, we did not conduct too much research on this localization method. Our future research will focus on solving the problem of dual partial discharge sources localization using a smaller number of sensors. We are currently exploring two technical routes concurrently. One team is studying ways to reduce the number of array elements in the sensor array, potentially sacrificing some precision to achieve a reduction in sensor count. The other team is searching for novel localization methods. We look forward to developing more practical and acceptable localization techniques that will be welcomed by transformer manufacturers and users.

## 5. Conclusions

This paper proposes a novel method for detecting and localizing dual PD sources inside transformers using a pressure-balanced fiber-optic ultrasonic sensor array with a regular tetrahedron structure and a direction-finding cross-localization algorithm. The sensor can be installed deep in the transformer oil, and its pressure-balanced structure design can reduce the obstruction of oil pressure on the diaphragm vibration, enabling it to capture the discharge signals from dual partial discharge sources. For scenarios where there are no obstacles blocking the discharge of dual PD sources, this method can achieve localization of dual PD sources inside transformer oil using only two sensor arrays.

When dual partial discharge sources occur in the oil channel of the winding, the ultrasonic signals generated by PD will undergo attenuation and distortion. In such cases, the detected waveforms can only be used for qualitative partial discharge detection and cannot accurately locate the fault point. If the periodic amplitude of the direct wave contained in the partial discharge ultrasonic signal is not attenuated, such as when it occurs in the interturn of the transformer winding, then its waveform can be used for localization. Otherwise, the position of the sensors needs to be adjusted to reduce the attenuation of the direct wave period caused by obstacles.

This detection method has a high dependence on sensor sensitivity and the direct wave of partial discharge. In the subsequent research work, different coating processes will be tried to reduce the diaphragm thickness and improve the sensor sensitivity. In addition, sensors will be installed at different positions of the transformer to allow the ultrasonic signals of partial discharges from different fault areas to directly reach the sensor array, avoiding obstruction. Furthermore, the localization method needs to be improved, using a smaller number of sensors to achieve more precise localization.

## Figures and Tables

**Table 1:** The summary table of technical changes for this section.

Unresolved Issues	Solution	Significance
The transformer oil pressure impedes the sensor diaphragm’s response, preventing the detection of ultrasonic signals from dual partial discharge sources.	Sensor chamber drilling	Allowing transformer oil to enter and achieve pressure balance
	Silica diaphragm coating	To improve the reflectivity of the reflective end face and reduce the impact of transformer oil entering.
	Fiber optic end face coating	

**Table 2:** Results of localization for dual PD sources in transformer oil.

Number	PD Source Coordinates/cm	Localization Results/cm	Distance Error/cm
1	(47, 48, 47)	(42.7, 58.8, 41.2)	12.9
	(50, 27, 53)	(48.2, 21.1, 48.6)	7.6
2	(70, 40, 49)	(85.1, 47.1, 55.1)	17.7
	(50, 57, 53)	(41.9, 66.2, 48.3)	13.1
3	(30, 41, 50)	(27.3, 47.2, 45.1)	8.4
	(75, 47, 56)	(68.7, 56.3, 67.2)	15.9
4	(40, 26, 40)	(37, 34, 52)	14.7
	(44, 38, 51)	(51, 45, 47)	10.7

**Table 3:** 35 kV single-phase transformer winding model dimensions.

Terms	Size (cm)
Outer diameter of the outer winding	50
Inner diameter of the outer winding	38
Outer diameter of the inner winding	26
Inner diameter of the inner winding	20
Winding height	60

**Table 4:** Results of localization for dual PD sources in transformer winding interturn.

Number	PD Source Coordinates/cm	Localization Results/cm	Distance Error/cm
1	(43, 170, 25)	(49.4, 185.6, 30.7)	17.8
	(54, 168, 30)	(60.7, 162.2, 38.1)	12.0
2	(52, 167, 45)	(61.2, 171.3, 39.8)	11.4
	(59, 174, 30)	(65.2, 169.1, 48.1)	19.7
3	(39, 176, 25)	(34.2, 170.3, 29.3)	9.4
	(58, 172, 45)	(62.3, 176.7, 40.3)	7.9
4	(42, 171, 35)	(49.1, 164.4, 42.9)	12.5
	(48, 166, 40)	(41.6, 176.2, 31.4)	14.8
5	(52, 167, 25)	(61.2, 175.2, 35.1)	15.9
	(58, 172, 40)	(51.2, 180.2, 31.9)	13.4

## Data Availability

The data supporting the results presented in this article are available upon request.
